# Statistical analysis of differential gene expression relative to a fold change threshold on NanoString data of mouse odorant receptor genes

**DOI:** 10.1186/1471-2105-15-39

**Published:** 2014-02-04

**Authors:** Evelien Vaes, Mona Khan, Peter Mombaerts

**Affiliations:** 1 Max Planck Research Unit for Neurogenetics, Max-von-Laue-Strasse 3, 60438 Frankfurt, Germany

**Keywords:** Gene expression studies, Statistical test, Differential gene expression, Fold change criterion, NanoString

## Abstract

**Background:**

A challenge in gene expression studies is the reliable identification of differentially expressed genes. In many high-throughput studies, genes are accepted as differentially expressed only if they satisfy simultaneously a p value criterion and a fold change criterion. A statistical method, TREAT, has been developed for microarray data to assess formally if fold changes are significantly higher than a predefined threshold. We have recently applied the NanoString digital platform to study expression of mouse odorant receptor genes, which form with 1,200 members the largest gene family in the mouse genome. Our objectives are, on these data, to decrease false discoveries when formally assessing the genes relative to a fold change threshold, and to provide a guided selection in the choice of this threshold.

**Results:**

Statistical tests have been developed for microarray data to identify genes that are differentially expressed relative to a fold change threshold. Here we report that another approach, which we refer to as tTREAT, is more appropriate for our NanoString data, where false discoveries lead to costly and time-consuming follow-up experiments. Methods that we refer to as tTREAT2 and the running fold change model improve the performance of the statistical tests by protecting or selecting the fold change threshold more objectively. We show the benefits on simulated and real data.

**Conclusions:**

Gene-wise statistical analyses of gene expression data, for which the significance relative to a fold change threshold is important, give reproducible and reliable results on NanoString data of mouse odorant receptor genes. Because it can be difficult to set in advance a fold change threshold that is meaningful for the available data, we developed methods that enable a better choice (thus reducing false discoveries and/or missed genes) or avoid this choice altogether. This set of tools may be useful for the analysis of other types of gene expression data.

## Background

Multiplex gene expression studies that are based on microarrays and next-generation sequencing result in the generation of large datasets. The simultaneous analysis of the expression of thousands of genes in these high-throughput approaches challenges statistical methods and data interpretation. Traditional statistical tests and analysis tools are therefore often insufficient.

One of the most popular types of biological experiments is a two-sample comparison. Gene expression studies often seek to identify genes that are Differentially Expressed (DE) between RNA samples from two types of biological conditions, such as gene knockout mice compared to wild-type mice. DE genes can give insights into biological mechanisms or pathways, and form the basis for further experiments. The traditional statistical method for identifying DE genes between two samples is the student’s t-test. But a microarray comparative experiment is faced with simultaneously assessing a large number of genes based on a small number of biological or technical replicates. Assessing such a large dataset with a small sample size challenges statistical methods: dealing with many genes but few replicates may lead to large Fold Changes (FC) driven by outliers, and to small error variances [1,2]. In order to overcome such problems, the t-test has been modified for microarray data analysis. The general idea behind these modifications is to obtain more stable estimates of the error variance of a given gene by “borrowing” information from all available genes. This goal is often obtained by applying (empirical) Bayesian methods. Examples of these modified tests for microarray data are the SAM test [3], the regularized t-test [3,4], and the B-statistic [1,5], as reviewed [2,6,7].

Other statistical approaches for microarray data analysis have introduced linear models [2,8-10]. These models allow for more flexibility, for instance when comparing more than two samples or introducing additional sources of variation. Such an example is Lin et al. [11], where a complex experimental design and research goal are addressed by setting various contrasts in the design of the linear model. The bioconductor package limma, developed by Smyth [12], applies a gene-wise linear model, and allows for the analysis of complex experiments (comparing many RNA samples), as well as more simple replicated experiments with two RNA samples. The package has further developed the ideas of Lönsstedt and Speed [1], which have been reset within the framework of linear models in order to address the challenges of microarray data analysis. The statistic (applied in limma) to identify DE genes is referred to as the moderated t-statistic, denoted by t* [5,12]. In the calculation of t*, shrinkage of the estimated error variances towards a pooled estimate is obtained through an empirical Bayes approach.

For certain biological problems, it is important to rank genes according to their FC or to impose on a gene to attain a predefined FC threshold before calling it DE. In such situations there remains often a disconnect between the concepts of a statistically significant differential expression (based on the p value) and a biologically meaningful differential expression (FC higher than a predefined threshold value). Even the modified tests may result in genes with small FCs to be considered statistically significant.

In order to integrate these statistical and biological concepts, a gene can be defined as DE when it satisfies a p value and a FC criterion [9,13-15]. The advantage of this combined approach is that the t-test or any of the modified tests can be combined with an ad-hoc FC criterion. The disadvantage of an ad-hoc FC criterion is that it does not take into account error variance and therefore offers no statistical confidence about future results. In addition, depending on the choice of the FC criterion and significance criterion, various interpretations of the same dataset are possible [16]. The moderated t-statistic [5] has been extended by McCarthy and Smyth into a new “test relative to a FC threshold”, abbreviated TREAT [17]. This method assesses formally whether the true differential expression is greater than a predefined FC criterion. TREAT offers greater specificity and reproducibility in identifying DE genes, compared to the combined approach of statistical test and ad-hoc FC criterion. TREAT has been also added to the limma package.

We have previously applied the NanoString digital platform [18] to study the expression of odorant receptor (OR) genes in mice [19,20]. In contrast to microarray data, where analog levels of fluorescent intensity are measured, NanoString data represent digital readouts of single molecules in the form of probe counts. These probes contain unique fluorescent bar codes, and RNA abundance of up to 800 genes can be analyzed in a single reaction in a single tube. The NanoString technology thus places itself between qPCR and microarrays in terms of throughput level [21]. We have analyzed with NanoString the expression of half of the OR gene repertoire of ~1200 genes [19]. Here, we have explored statistical tools for our NanoString data, and developed a systematic approach for identifying DE genes with respect to a given FC threshold.

We explored the moderated t-statistic (t*), the derivation of which was driven by microarray data (high throughput), versus the classical t-statistic on comparative NanoString experiments (medium throughput). We found that t* does not show a protective effect (i.e. fewer false discoveries) over t on our NanoString data. But we also wanted to test whether differential expression is greater than a FC threshold. Therefore we used the analysis relative to a FC threshold together with the classical t-statistic in two approaches. The first approach is similar to TREAT as published for t* [17], and we refer to it as tTREAT. Then we addressed the arbitrary choice of the FC criterion itself, and developed a two-stage approach, tTREAT2. We describe the performance of TREAT, tTREAT, and tTREAT2 on our NanoString data, both in data simulation experiments and on biological data.

The variability of the FC of a gene is inversely related to the expression level of that gene; lowly expressed genes tend to have a greater error in their measured FC levels [4,22]. These lowly expressed genes can thus more easily reach a certain FC threshold, and the inverse is true for highly expressed genes. A non-linear model, the Limit Fold Change model (LFC), has been developed to identify DE genes based on this relation [22]. We have applied a similar LFC model on our NanoString data. We did not use the LFC model as a tool for identifying DE genes but to set appropriate FC thresholds for genes with various ranges of expression levels, in order to avoid a subjective choice of FC criterion. The FC thresholds that we thus derived were then used in a subsequent analysis relative to a threshold in order to identify the DE genes. We refer to this setup as the running FC model, and illustrate its use and benefit on biological data.

## Results

### Biological data of odorant receptor gene expression

The main olfactory epithelium is located in the nasal cavity of the mouse, and detects volatile chemicals (odorants) in the inhaled air. The sense of smell must detect chemical stimuli with an immense variety in physicochemical properties. To accommodate this broad recognition, the mammalian olfactory system has evolved a large repertoire of molecular receptors, odorant receptors. These receptors are expressed by olfactory sensory neurons in the main olfactory epithelium. In the mouse, ~1200 odorant receptors are encoded by distinct genes, which form the largest gene family in its genome. It is widely accepted that a mature olfactory sensory neuron expresses only one of the ~1200 odorant receptor genes. The molecular and genetic mechanisms that regulate this one receptor - one neuron rule remain unclear, and are the focus of our research. We have demonstrated the role of the H element [23] and the P element [24] in the regulation of expression of clusters of odorant receptor genes by genetically engineering mouse strains that lack the H element [25] or the P element [19]: these are the ΔH and ΔP strains and the ΔHxΔP double knockout strain [19]. Another mouse strain is the ΔOlfr7Δ strain [19]; by means of chromosome engineering [26], we excised a 2.4 megabase region on Chromosome 9 that contains the *Olfr7* cluster consisting of 99 OR genes [27]. We have also characterized temporal expression patterns of 531 odorant receptor genes in adult and aged mice [20].

Here we used datasets [19] from a NanoString analysis of 558 OR genes comparing knockout versus wild-type mouse strains. Specifically, we used NanoString data obtained from six mutant mice of the ΔHxΔP strain (cartridge MK29) compared to 12 control (wild-type) mice (cartridges MK29 and MK37, six mice each); these 18 mice are in a mixed genetic background, C57BL/6 J × 129/SvEv. Another dataset was obtained from six mice of the ΔOlfr7Δ strain, compared to six control (wild-type) mice (cartridge MK38); these 12 mice are in pure genetic background, 129/SvEv. With NanoString CodeSet Gorilla, we determined the RNA abundance for 558 OR genes from 1 μg RNA of whole olfactory mucosa tissue samples. Each lane of a NanoString cartridge represents a different RNA sample and mouse. Thus, there are 6–12 biological replicates per biological condition, and no technical replicates.

### Approaches relative to a FC threshold

Our novel method tTREAT is similar to TREAT [17]. It is applied to the regular student’s t-statistic, and requires a predefined FC threshold *τ*. For the two-stage design, tTREAT2, a second threshold *θ*, with *θ*>*τ*, is used in a first “stop and go” stage in which it is decided whether a gene is non-DE (stop) or whether it can proceed to the next stage (go). Then, for all the “go” genes, a tTREAT test with FC threshold *τ* is applied. In the running FC model, a non-linear model for FC versus average gene expression is first used to determine various FC thresholds *τ*_*i*_ for a number of ranges of expression levels. Genes are then binned in k gene expression levels, and the appropriate *τ*_*i*_ is used per concentration bin in a subsequent analysis relative to a FC threshold.

### Simulated data

Because NanoString data are not yet as widely studied as microarray data, we have conducted a data simulation procedure that represents one of our typical two-group comparison NanoString experiments.

The procedure for simulating one NanoString dataset was conducted according to the following steps and distributional assumptions:

• To get a general idea about the variances of NanoString gene expression data, the genes in the ΔHxΔP dataset were used as an example gene population. Biological data from ΔHxΔP mice were chosen, as they represent a noisier dataset due to the mixed genetic background of this strain [19]: the resulting simulated data will not represent the cleanest example. The ΔHxΔP dataset was used only for the next step of the simulation exercise.

• Subsequently, 100 real variances across genes, $\sigma_{g}^{2}$, were drawn from an inverse-gamma distribution: *Inv* − *Gamma*(*g*_1_, *g*_2_). The parameters *g*_1_ and *g*_2_ are estimated by a Maximum Likelihood procedure in the above described gene population.

• Each of the 100 $\sigma_{g}^{2}$ gave rise to three randomly drawn real differences *β*_*g*_. The $\sigma_{g}^{2}$ and three corresponding *β*_*g*_ were included in one of the following three gene groups:

(Group 1) DE genes: The *β*_*g*_ were drawn from a Gaussian $\sim N\left(0,\sigma_{g}v_{\mathrm{start}}\right)$ and had to satisfy the criterion: |*β*_*g*_| ≥ *log*_2_(*ω*).

(Group 2) Non-DE genes, noisy: Similarly, *β*_*g*_  drawn from $\sim N\left(0,\sigma_{g}v_{\mathrm{start}}\right)$ but with |*β*_*g*_| < *log*_2_(*ω*).

(Group 3) Non-DE genes, regular: Obviously for this group the real *β*_*g*_ were set to 0.

Note that empirically, the normal distribution seems acceptable for the *β*_*g*_, based on our NanoString data.

• The *β*_*g*_ that were produced in the previous step served as true differences that were then subsequently used to simulate three possible estimates ${\hat{\beta }}_{g}$ from a Gaussian $\sim N\left(\beta_{g},\sigma_{g}\sqrt{z_{g}}\right)$. Similarly residual variances were drawn from a Chi-square distribution: $\sim \frac{\sigma_{g}^{2}}{df_{g}}\chi^{2}\left(df_{g}\right)$

Since the quantity *ω* used to initiate the simulation as described above defines the DE genes by the rule |*β*_*g*_| ≥ *log*_2_(*ω*), the distributional center of the ${\hat{\beta }}_{g}$ of DE genes actually lies a little further than the actual value of *ω*, depending on the value of their variance: $\sigma_{g}\sqrt{z_{g}}$. Henceforth, we will refer to *ω* as the FC *ω* with respect to which the data was simulated.

Every such simulated dataset was thus initially based on 100 $\sigma_{g}^{2}$ leading to a total of 900 genes. The regular non-DE genes (group 3) were fixed as 20% of the 900 genes. However, to assess whether the number of DE genes in a dataset influences the results, the percentage of DE genes (and thus also the percentage of the noisy non-DE genes) was varied between 1% and 40% (noisy non-DE genes: 79% and 40%, respectively).

On the simulated data, the following statistical approaches for identifying genes that are DE with regard to a certain FC threshold were assessed:

(1) TREAT with regard to FC threshold *τ*

(2) tTREAT with regard to FC threshold *τ*

(3) tTREAT2 with a bilateral p value calculation in the second stage with regard to FC thresholds *θ* (stage 1) and *τ* (stage 2)

As the two-stage design tTREAT2 relies on the choice of two related FC thresholds, one for each of the two stages, it is not straightforward to compare the performance of tTREAT2 to TREAT and/or tTREAT. Therefore we have derived various experimental schemes to show in which situations the use of a two-stage design like tTREAT2 can be beneficial.

### Results on simulated data

We simulated 400 realizations of a two-group comparison NanoString experiment with 900 genes for which the DE genes are simulated with respect to a certain FC difference *ω* as described above. As such we know beforehand to which group (DE or non-DE) each gene in these 400 datasets belongs. We can therefore assess the performance of the procedures described in the methods section. For this purpose, we fix *v*_*start*_ = 8 and the significance (type I error) was set at 0.05 or at 0.01. The mean of the false discoveries (false positives) and missed genes (false negatives) over the 400 generated datasets is plotted for the various statistical approaches for FCs *ω* (the FC difference with respect to which the data are simulated) and *τ* (the actual FC threshold used in the testing procedure). We also report mean Area Under the ROC-curve (AUC) values over the 400 generated data sets.

**Figure 1:**
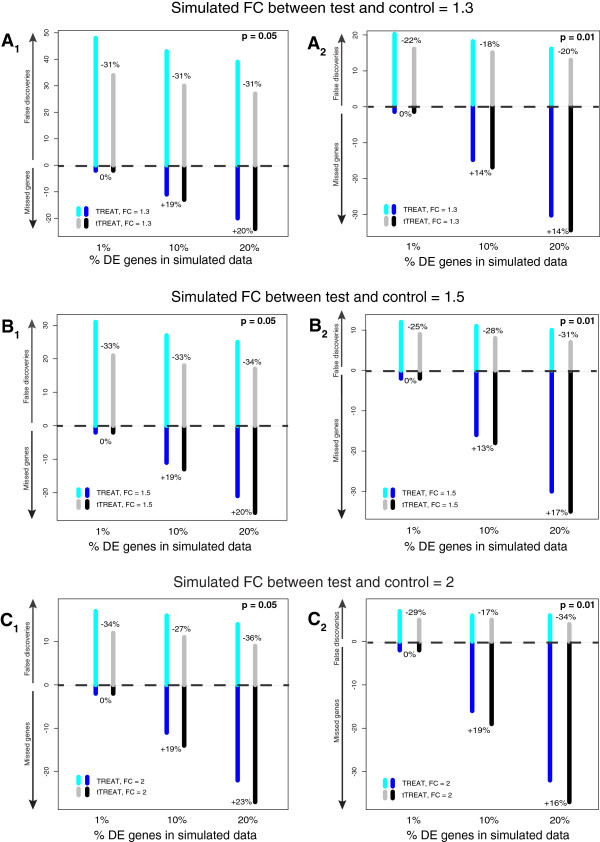
**False discoveries and missed genes for TREAT and tTREAT on simulated data, at p = 0.05 or at p = 0.01. (A**_**1**_**)** The positive y axis shows the average of the false discoveries, and the negative y axis shows the average of the missed genes for TREAT and tTREAT on 400 simulated datasets. The x axis shows the percentage of DE genes (1%, 10% and 20%) that is simulated in each case. The data are simulated with respect to a FC difference *ω* of 1.3 (up or down), and the FC threshold *τ* used for TREAT and tTREAT is also 1.3. The percentages next to the gray and black bars of tTREAT represent the percentual decrease (prefixed with a minus sign) or increase (prefixed with a plus sign) in false discoveries or missed genes with respect to the reference, TREAT (depicted in cyan and blue). The significance cut-off was set at p = 0.05. **(A**_**2**_**)** Same conditions as in panel **A**_**1**_, except that significance was set at p = 0.01. **(B**_**1**_**)** Similar to panel **A**_**1**_, but now the data are simulated with respect to a FC difference *ω* of 1.5, and the FC threshold *τ* used for TREAT and tTREAT is also 1.5. The significance cut-off was set at p = 0.05. **(B**_**2**_**)** Same conditions as in panel **B**_**1**_, except that significance was set at p = 0.01. **(C**_**1**_**)** Similar to panel **A**_**1**_, but now the data are simulated with respect to a FC difference *ω* of 2, and the FC threshold *τ* used for TREAT and tTREAT is also 2. The significance cut-off was set at p = 0.05. **(C**_**2**_**)** Same conditions as in panel **C**_**1**_, except that significance was set at p = 0.01.

Figure 1 and Additional file 1 compare TREAT and tTREAT on a simulation exercise where the FC difference *ω* and the FC threshold *τ* were chosen to be equal. We tested values for *ω* = *τ* of 1.3 (Figure 1A_1_ and 1A_2_), 1.5 (Figure 1B_1_ and 1B_2_) and 2 (Figure 1C_1_ and 1C_2_). Across the various percentages of DE genes (indicated on the x axis), the percentages next to the gray and black bars of tTREAT in Figure 1 represent the percentual decrease (prefixed with a minus sign) or increase (prefixed with a plus sign) in false discoveries or missed genes with respect to the reference, TREAT. We find that tTREAT always results in fewer false discoveries than TREAT, at p = 0.05 and p = 0.01. Moreover, when p = 0.05 is applied as a significance cut-off (Figure 1A_1,_1B_1_ and 1C_1_), tTREAT decreases the false discoveries for only a few more missed genes compared to TREAT (Additional file 2). For p = 0.01 (Figure 1A_2,_1B_2_ and 1C_2_) the latter is true for datasets with small percentages of DE genes but for datasets with ~10% of DE genes, the decrease in false discoveries is nullified by the increase in missed genes (Additional file 2). In terms of AUC performance there is no difference between TREAT and tTREAT across the percentages of DE genes (Table 1).

**Table 1:** Area under the ROC curve (AUC) for various methods on simulated data

**AUC results completing Figure**1				
***Simulated FC between test and control = 1.3***				
		1% DE genes	10% DE genes	20% DE genes
TREAT, FC = 1.3	AUC* [95%PI]	96.1 [95.6-96.7]	96.3 [96.2-96.5]	96.4 [96.3-96.6]
tTREAT, FC = 1.3	AUC* [95%PI]	95.9 [95.3-96.4]	96.2 [96.1-96.3]	96.2 [96.1-96.3]
***Simulated FC between test and control = 1.5***				
	1% DE genes	10% DE genes	20% DE genes	
TREAT, FC = 1.5	AUC* [95%PI]	97.3 [96.8-97.7]	97.3 [97.2-97.5]	97.3 [97.2-97.4]
tTREAT, FC = 1.5	AUC* [95%PI]	97.0 [96.5-97.5]	97.0 [96.8-97.1]	96.9 [96.8-97.0]
***Simulated FC between test and control = 2***				
		1% DE genes	10% DE genes	20% DE genes
TREAT, FC = 2	AUC* [95%PI]	98.0 [97.7-98.4]	98.2 [98.1-98.3]	98.2 [98.1-98.3]
tTREAT, FC = 2	AUC* [95%PI]	97.3 [96.8-97.8]	97.5 [97.3-97.6]	97.5 [97.3-97.6]
**AUC results completing Figure** 2				
***Simulated FC between tets and control = 1.5 and Stringent test***				
		1% DE genes	10% DE genes	20% DE genes
tTREAT, FC = 1.5	AUC* [95%PI]	96.7 [96.2-97.2]	97.0 [96.9-97.2]	97.0 [96.8-97.1]
tTREAT, FC = 2.5	AUC* [95%PI]	91.1 [89.5-92.7]	93.7 [93.5-94.0]	93.7 [93.5-94.0]
tTREAT2, FC = 2.5/1.5	AUC* [95%PI]	96.7 [96.1-97.3]	97.3 [97.1-97.4]	97.2 [97.1-97.4]
**AUC results completing Figure** 3				
***Simulated FC between test and control = 2.5 and non-stringent test***				
		1% DE genes	10% DE genes	20% DE genes
tTREAT, FC = 2.5	AUC* [95%PI]	97.6 [97.2-98.1]	97.6 [97.4-97.7]	97.6 [97.5-97.7]
tTREAT, FC = 1.5	AUC* [95%PI]	97.7 [97.4-98.0]	97.7 [97.6-97.8]	97.8 [97.7-97.8]
tTREAT2, FC = 2.5/1.5	AUC* [95%PI]	96.7 [96.3-97.1]	96.7 [96.6-96.9]	96.7 [96.6-96.8]

*Mean AUC over the 400 simulated data sets with 95% prediction intervals.

**Figure 2:**
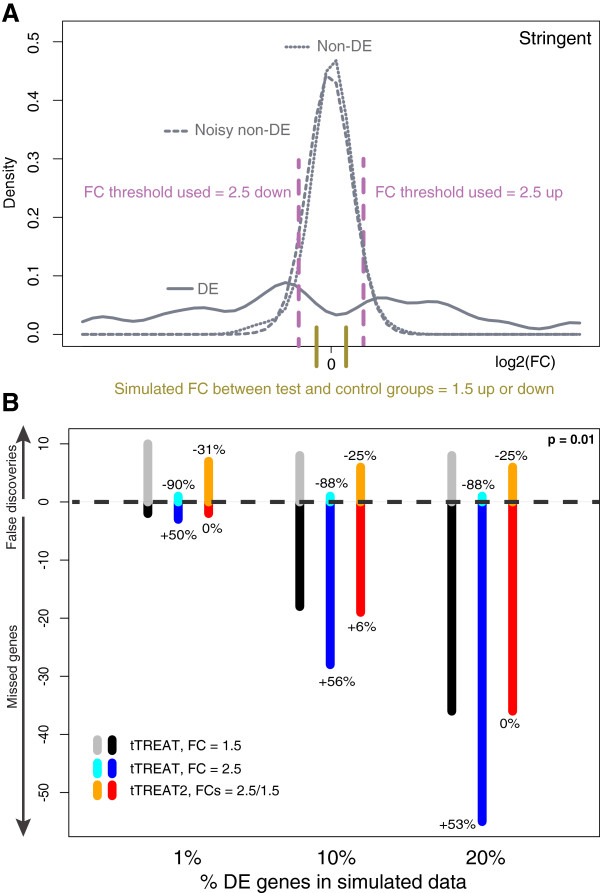
**The two-stage design in a stringent test situation. (A)** Data simulation experiment: empirical density functions of the DE genes (solid curve), noisy non-DE genes (dashed curve), and non-DE genes (dotted curve). The vertical olive lines indicate the simulated FC difference *ω* between test and control groups. The choice of *ω* determines the peak of the density function of the DE genes, which lies a little further. The non-DE genes are distributed around a FC difference of 0. A stringent test relative to a FC threshold *τ*, as indicated by the purple dashed lines, identifies only the DE genes to the right or left of these lines.** (B)** The positive y axis shows the average number of false discoveries, and the negative y axis shows the average number of missed genes, over 400 generated datasets for three tests relative to a FC threshold. The x axis shows the percentage of DE genes (1%, 10% and 20%) that is simulated in each case. Significance is set at p = 0.01. The data are simulated with respect to a FC difference *ω* of 1.5. The reference tTREAT with a FC threshold *τ* of 1.5, thus a test with *τ* = *ω*, is depicted in gray and black. The stringent test with a FC threshold *τ* of 2.5 is in cyan and blue. The decrease (prefixed with a minus sign) or increase (prefixed with a plus sign) in false discoveries or missed genes with respect to the reference (tTREAT with *τ* of 1.5) is indicated above or below the respective colored bar. When applying tTREAT2 (orange and red) the false discoveries are decreased with regard to the reference test, but the missed genes are not as much increased with regard to the reference test as is the case with the stringent test.

In order to illustrate the added value of the safety margin around the FC threshold *τ* that has been chosen for statistical analysis, two simulation experiments were done. In the first experiment, the data were simulated such that the actual DE genes had a FC difference *ω* of at least 1.5 (up or down), and p was set at 0.01. A blind, stringent tTREAT test at the higher FC threshold *τ* of 2.5 (Figure 2A) will result in a high number of missed genes (dark blue bars, Figure 2B). The reference test for this scenario is a tTREAT with FC threshold *τ* = *ω* = 1.5, represented by the black and gray stacked bars in Figure 2B. (See Additional file 3A for stacked bars across 40 percentages of DE genes). The stringent tTREAT (*τ* of 2.5) decreases, as expected, the false discoveries, by 90% in comparison to the reference test (cyan vs gray bars, Figure 2B) but at the cost of a 50% increase in missed genes (dark blue vs black bars, Figure 2B). When a two-stage approach is used with FCs *θ* and *τ* chosen at 2.5 and 1.5, the high number of missed genes of the blind, stringent tTREAT test (with FC threshold *τ* of 2.5) is reduced (red vs dark blue bars, Figure 2B). At the same time, the two-stage approach has a protective effect over the reference tTREAT test (FC *τ* = 1.5) by decreasing the number of false positives (orange vs grey bars, Figure 2B). The stringent test has a much lower AUC than the reference test and two-stage approach (Table 1). The two-stage approach has the best overall performance (highest AUC value) for this experiment.

**Figure 3:**
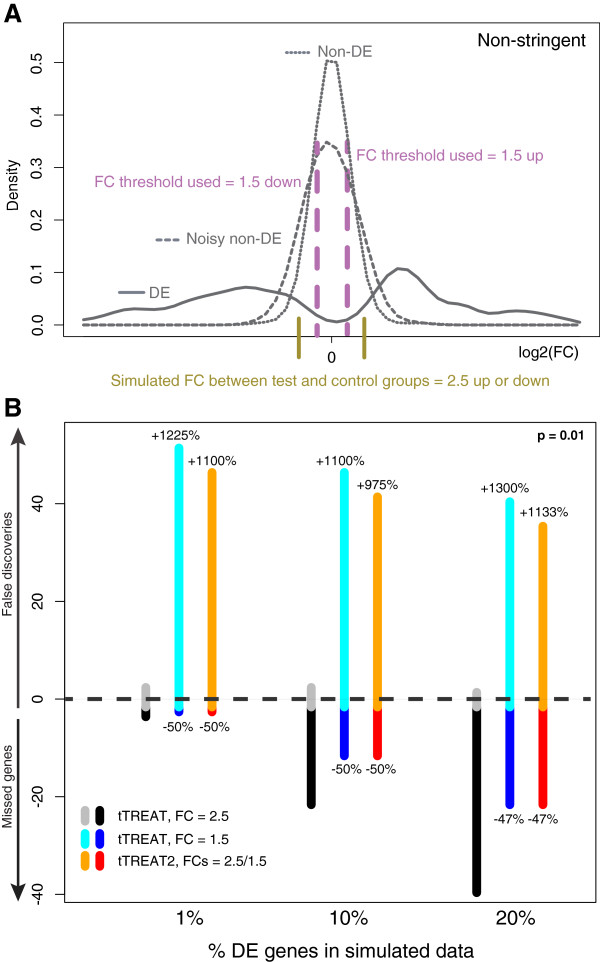
**The two-stage design in a non-stringent test situation. (A)** Data simulation experiment: empirical density functions of the DE genes (solid curve), noisy non-DE genes (dashed curve), and non-DE genes (dotted curve). The vertical olive lines indicate the simulated FC difference *ω* between test and control groups. The choice of *ω* determines the peak of the density function of the DE genes, which lies a little further to the right. The non-DE genes are distributed around a FC difference of 0. A non-stringent test relative to a FC threshold *τ*, as indicated by the purple dashed lines, identifies all the DE genes to the right or left of these lines, but also many non-DE genes.** (B)** The positive y axis shows the average number of false discoveries, and the negative y axis shows the average number of missed genes, over 400 generated datasets for three tests relative to a FC threshold. The x axis shows the percentage of DE genes (1%, 10% and 20%) that is simulated in each case. Significance is set at p = 0.01. The DE genes are simulated with respect to a FC difference *ω* of 2.5. Here, the reference test is tTREAT with a FC threshold *τ* of 2.5, thus a test with *τ* = *ω* (gray and black). The non-stringent test with a FC threshold *τ* of 1.5 is in cyan and blue. When using a safety margin in tTREAT2 (orange and red), the number of false discoveries increases much with regard to the reference test (tTREAT with *τ* = 2.5), as marked by the positive percentage above the orange bar. This increase is not as large as is the case with the non-stringent test. The negative percentages underneath their respective bars show the decrease in missed genes.

A second simulation experiment focuses on the inverse situation. Data are simulated with respect to a FC of *ω* = 2.5 (up or down), and p = 0.01. One tTREAT test aims too low with a FC threshold of 1.5 (purple dashed lines in Figure 3A), leading to many false discoveries. The reference test for this scenario is a tTREAT with FC threshold *τ* = *ω* = 2.5, represented by the black and gray stacked bars in Figure 3B. (See Additional file 3B for stacked bars across 40 different percentages of DE genes). A two-stage approach decreases the number of false positives that are obtained by the non-stringent test with too low a FC threshold (orange vs light blue bars, Figure 3B) while maintaining the false negatives (red vs dark blue bars, Figure 3B). The AUC values show that the overall performance of these three tests is very similar (Table 1).

### Benefit of tTREAT2 for a biological dataset

**Figure 4:**
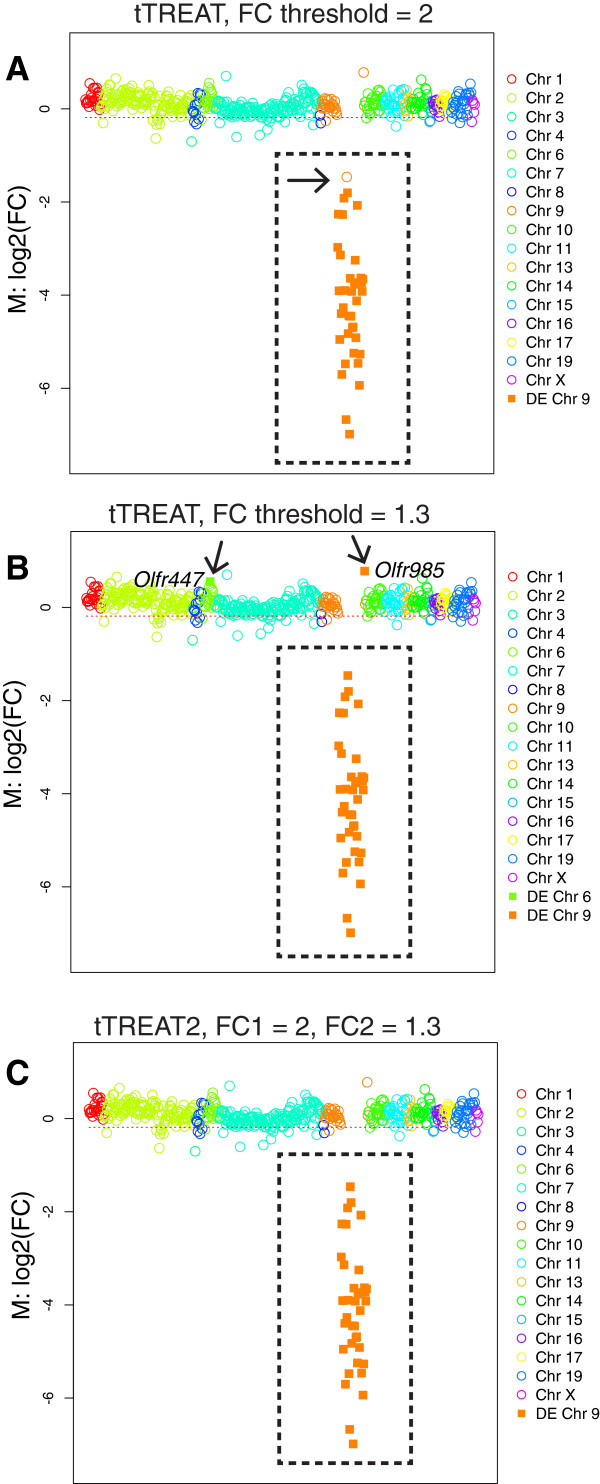
**The two-stage design on biological data: ΔOlfr7Δ mice. (A)** MC plot of 558 OR genes. Filled squares represent genes that are identified as DE by tTREAT with an FC threshold *τ* of 2, comparing ΔOlfr7Δ mutant mice to control (wild-type) mice. The black stippled rectangle encompasses the 37 genes in the *Olfr7* cluster deletion; these genes do not exist in the genome of ΔOlfr7Δ mice, and are thus obviously DE. The black arrow indicates a gene (*Olfr916*) in the *Olfr7* cluster deletion that was missed, thus that was identified as non-DE. Empty circles represent non-DE genes. **(B)** MC plot of 558 OR genes. Filled squares represent genes that were identified as DE by tTREAT with a FC threshold *τ* of 1.3. The black stippled rectangle encompasses the 37 genes in the *Olfr7* cluster deletion. The black arrows indicate two OR genes that were identified as DE, but reside outside of the *Olfr7* cluster deletion, and therefore could be false discoveries. Because *Olfr985* is close to the genomic deletion, its expression could be affected. Empty circles represent non-DE genes. **(C)** MC plot of 558 OR genes. Filled squares represent genes that identified as DE by tTREAT2 with FC thresholds *θ* = 2 and *τ* = 1.3. The black stippled rectangle encompasses the 37 genes in the *Olfr7* cluster deletion. Empty circles represent non-DE genes. tTREAT2 now identifies the 37 genes in the deleted cluster as DE, and no other gene: there are no missed genes, and no false discoveries.

We analyzed samples of six ΔOlfr7Δ mutant mice in comparison to six control mice with our NanoString Gorilla CodeSet containing 558 OR genes. The *Olfr7* cluster contains 99 OR genes, and for 40 OR genes we could design specific probes. Of these 40 genes, 37 were defined as informative, as the median of the normalized NanoString counts in the control mice is at least 100. Because these 37 OR genes are not present in the genome of the mutant mice, the counts for these OR genes in mutant mice should be at background levels, and these genes are a priori DE. The ∆Olfr7∆ strain is thus the ultimate negative control for probe specificity. Because the maximum counts for the negative controls is ~50, we reasoned that even for expressed *Olfr7* cluster genes with low normalized counts (~130), we could expect a >2-fold change. We thus set the FC threshold *τ* at 2 in a first tTREAT analysis, with p = 0.01. The MC plot in Figure 4A shows the results: of the 37 genes in the deleted *Olfr7* cluster, 36 are detected as having a FC significantly lower than ½, but one deleted gene, *Olfr916*, is missed. We lowered the threshold to *τ* = 1.3 in a second tTREAT analysis: a total of 39 genes is now identified with a FC significantly lower than 1/1.3 or higher than 1.3 (Figure 4B). But two genes with a FC higher than 1.3 turn out to reside outside the deleted cluster: *Olfr985*, which could perhaps make sense given it resides close to the deleted region, and *Olfr447*, which is on chromosome 6 and ought not to be affected. Importantly, when we apply a two-stage approach, tTREAT2 with *θ* = 2 and *τ* = 1.3, the 37 genes of the deleted cluster, and only these genes, are found to be DE (Figure 4C): no genes are missed, and no genes outside the cluster are identified as DE. The experimentally rare case of a deletion mutant, in which certain genes are not present, thus enables us to demonstrate the benefit of tTREAT2.

### The running FC model

**Figure 5:**
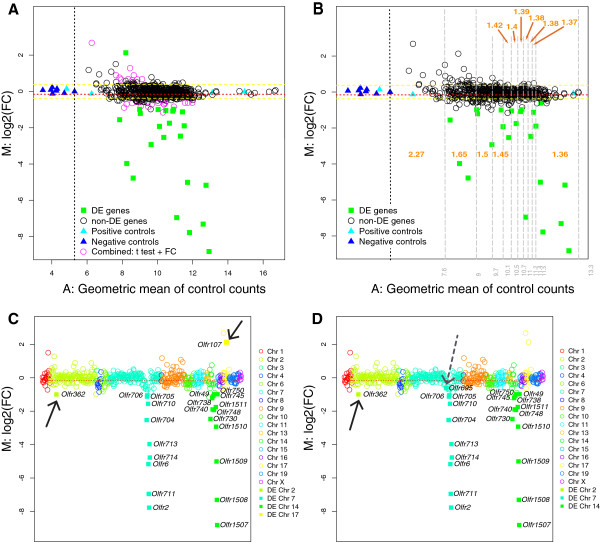
**Running fold change model on biological data: ΔHxΔP mice. (A)** MA plot of 558 OR genes. Filled green squares represent genes that were identified as DE by tTREAT with a FC threshold of 1.5, comparing ΔHxΔP double mutant mice to control (wild-type) mice. Empty circles represent non-DE genes. The empty pink circles indicate genes that satisfy the combined criteria of a significant p value for a regular t-test (p < 0.01) and a FC value >1.5 or <1/1.5. The black vertical stippled line indicates the maximum of the negative controls. The red horizontal stippled line is the mean of the M values. The yellow stippled lines are equal to M values that represent 1.3 fold up or down: ± *log*_2_(1.3). **(B)** MA plot of 558 OR genes. Filled green squares represent genes that were identified as DE by a running FC model based on tTREAT. Empty circles represent non-DE genes. The grey vertical stippled lines delineate ranges of gene expression, for which various FC thresholds have been used. Orange numbers indicate the FC thresholds that are applied in the subsequent tTREAT analysis. **(C)** MC plot corresponding to the results shown in panel A. M values are arranged according to the relative gene order along the chromosomes, which are indicated in various colors. The black arrows indicate two OR genes that have been identified as DE, but reside outside of the H and P clusters; these are most likely false discoveries. **(D)** MC plot corresponding to the results shown in panel **C.** The solid black arrow indicates an OR gene that has been identified as DE, but resides outside of the H and P clusters, and is most likely a false discovery. The dashed black arrow indicates an OR gene that is identified as DE and resides within the P cluster; this gene is missed in tTREAT.

We applied the running FC model on our NanoString data obtained from six ΔHxΔP mutant mice and 12 control mice. As these mice are from a mixed genetic background, we used a less-stringent tTREAT with *τ* = 1.5, and p = 0.01. The results are shown as an MA plot in Figure 5A and as an MC plot in Figure 5C. We find that nine genes from the P cluster and 12 genes from the H cluster have a FC significantly lower than 1/1.5. But two additional genes, *Olfr362* on Chromosome 2 and *Olfr107* on Chromosome 17, are identified as DE by tTREAT with *τ* = 1.5. When we apply a running FC model, a FC threshold value *τ* is not chosen. Instead the model calculates a different *τ* for ten gene expression levels. These values are ≥1.5 for the lower expression levels and drop to 1.36 for the very high expression levels (Figure 5B). After calculating the *τ* values, we applied them together with a tTREAT test in our running FC model. This model identified 23 DE genes, 22 of which were also identified by tTREAT with *τ* = 1.5 (Figure 5D). The lowly expressed gene *Olfr107* (on Chromosome 17) is no longer identified as DE by the running FC model, but the model identified an additional OR gene, *Olfr695*, which is plausible as it resides within the P cluster.The advantage of tests relative to a FC threshold over combined approaches (statistical test outcome + FC criterion) is illustrated in Figure 5A. By combining the regular t-test and a FC criterion of 1.5, a total of 49 genes are identified as DE. Of these, 20 reside outside the H and P clusters (pink circles in Figure 5A), and are thus in reality most likely not DE.

## Discussion

Gene expression has been studied extensively for a wide variety of biological problems. Over the years various techniques have been developed for and applied to gene expression analysis. Ordered from low to high throughput, these techniques include qPCR, NanoString, microarrays, and RNA sequencing. These techniques come with their own specifications, advantages and disadvantages [21,28] but each also with specific challenges for statistical analysis. Microarrays have thus far been the most widely used approach to study gene expression, and have therefore led to numerous analytical tools and statistical tests. Many of these tools can also be applied to NanoString data or qPCR data, and some have resulted in practical software packages for NanoString [29,30].

The moderated t (referred to as t*) based TREAT, which was developed for microarray data [5,17] works well on our NanoString data. But we found that tTREAT with regular t is even more appropriate. Moreover, we propose an alternative approach that defines a safety margin around the FC threshold. This tTREAT2 application in two subsequent stages can be beneficial when data are expected to be noisy around a chosen FC threshold. We also developed a technique for finding FC thresholds that vary with the expression level of genes, followed by applying these objectively set thresholds in tTREAT. In principle, this running FC model can be applied to gene expression data obtained with any technique.

The goal of this study is not to determine which test has the highest performance on NanoString data, although we describe the overall performance (AUC values) of TREAT, tTREAT and tTREAT2 on simulated data. Statistical tests like TREAT, fine-tuned to gene expression data, are difficult to outperform. In certain situations, however, an alternative test may prove more beneficial for the technology that is used and the problem that is studied. In our studies of odorant receptor gene expression, a falsely discovered gene leads to extensive follow-up experiments such as in situ hybridization and even generation of a new mouse strain by gene targeting. Such follow-up experiments are costly, lengthy, and time-consuming. We thus need to minimize false discoveries. When using NanoString data, we find that tTREAT protects more against false discoveries at significance cut-offs of p = 0.05 and p = 0.01. It is perhaps counterintuitive that tTREAT instead of TREAT is more beneficial for our dataset, as TREAT and the moderated t statistic are known for their ability to decrease false discoveries in microarray experiments.

There are four main messages. First, we demonstrate with data simulations that tTREAT is more appropriate for our NanoString data than TREAT. We used biological data to initiate data simulation experiments, and find that tTREAT results in fewer false discoveries compared to TREAT when using significance cut-offs of p = 0.05 or p = 0.01. But when the percentage of DE genes in the simulated data is high, the advantage of tTREAT over TREAT in terms of false discoveries is countered by tTREAT missing more DE genes. When the overall performance of TREAT and tTREAT is compared by AUCs (thus independently of the chosen p value), there is no difference on our simulated data. The TREAT Bayesian approach shrinks (or blasts) the individual gene sample variances towards a pooled estimate, which works particularly well when the number of replicates (arrays) is small; often no more than two replicates are available for microarrays [5]. The NanoString counts in our dataset are not noisy and the throughput is much lower (558 genes) compared to a microarray experiment. So some of the gene-wise variances may be shrunk (or blasted) by too large a factor when applying TREAT on our NanoString data, as the hierarchical Bayesian model is expecting a few outlier variances.

Second, when noisy data are expected, with many non-DE genes showing signs of being increased or decreased, it is beneficial to use a safety margin around the chosen FC threshold in an analysis relative to a FC threshold. A good choice seems to set *θ* to *τ* + 0.5 or *τ* + 1.

Third, the particular choice of a FC threshold can influence the interpretation of biological data [16]. The running FC model can be applied in order to avoid the subjective choice of a FC threshold, or when data vary strongly with expression levels. We used the same dataset to determine the FC thresholds for various gene expression levels and to apply tTREAT, which may seem suboptimal. It would be ideal to determine FC thresholds on an independent dataset before using these thresholds in the subsequent analysis of the actual data. But often obtaining these independent data may be too expensive or impossible. We believe that performing the running FC model on the same dataset gives accurate results despite its data-driven nature in such circumstances.

Fourth, the choice of analytical tools must be driven by the research goals, the gene expression technique, and the hypothesis that is being tested. TREAT, tTREAT, or tTREAT2 either give a high number of false discoveries and few missed genes, only a few false discoveries for a higher number of missed genes, or an average but similar number of false discoveries and missed genes. The inevitable trade-off between false discoveries and missed genes must be evaluated dependent on the experimental context. In our case - NanoString studies of odorant receptor gene expression - we want to minimize the false discoveries, because the validation and follow-up experiments are laborious and expensive. But for other projects, the inverse may be desirable: the number of missed genes needs to be minimized, for instance in large-scale screening of molecules against a drug target, where missing a potential blockbuster drug cannot be afforded.

In the running FC model, the choice of the FC percentile in each bin in the development of the LFC model (step 1) as well as the choice of the k bins when applying the model (in step 3) affect the final results of the running FC model, thus the DE genes discovered. Depending on the percentage of DE genes that is expected, percentile values ranging from P55 (many DE genes are expected) to P95 (a few DE genes are expected) are deemed acceptable. Regarding the number of k bins, a good coverage of the full expression range of the genes under analysis should be ensured.

Performance results on a single simulated dataset (instead of 400) would have been subject to chance (in favor or disfavor of one method). Repeating the simulations 400 times permits an understanding of the variability in test performance that may be obtained between different simulations. The variability of our mean results is assessed by the 95% prediction intervals around the mean number of false discoveries (missed genes respectively) on these 400 data simulations. Neither of the statistical tests shows outliers in performance that could contradict our conclusions. Our data simulation procedure requires a “real” data input at the beginning only to get an idea about the distribution of gene error variances for NanoString. This input came from our data as described in the results. We also used an independent, published NanoString data set with ~250 mouse genes (cell-cycle and G2/M related functions) [31] to initiate the set of data simulation experiments again. We find that simulations initiated by these data lead to very similar results (data not shown).

We present approaches for testing the significance of gene expression relative to a FC threshold, with a focus on our NanoString data on OR genes. When carrying out these hundreds of significance tests simultaneously, the issue of multiple testing can be raised. A significance analysis for microarrays (SAM) test has been developed [3] that encompasses both a test statistic for ranking (a slightly tuned version of student’s t) and a way to control the False Discovery Rate and thus to adjust for multiple testing. The advantage of our approaches is that they provide p values that can be adjusted by a multiple testing method of choice, ranging from Bonferroni to Benjamini and Hochberg [32]. There is a wide variety of adjustment procedures for multiple testing. The choice of the most appropriate procedure for a given analysis is not straightforward. Guidelines and criteria to be attributed to the multiple testing approaches have been described [2]. In general, the formulation of a composite null-hypothesis forces TREAT, tTREAT, and tTREAT2 to rely on a more conservative way of p value calculation [17]. The distribution of the resulting p values is thus skewed towards the larger values, which has consequences: (1) if a method for adjusting multiple testing problems relies on the uniformity of the distribution of the p values, it cannot be used as this criterion is not met and (2) we feel that when applied to NanoString data (for which the technical maximum is 800 genes), these already conservative p values should not be adjusted by yet another conservative method.

For the development of TREAT and initially also for t* [5,17], it was decided to use gene-wise linear models with arbitrary coefficients and contrasts of interest (making them widely applicable) as a contextual and practical background; see also the limma package [12]. The tTREAT, tTREAT2, and the running FC model are also based on gene-wise linear models. Others [8] have used linear models, or, more precisely, analysis of variance models (ANOVA) to assess DE genes in microarray experiments. The main difference is that all genes are included in a single linear model, presenting the advantage that the normalization process is combined with the data analysis and thus pre-normalization of the data is not necessary. Obviously for such an ANOVA model to be justified, its underlying model assumptions should be met, but they can be assessed quite straightforward. For a smaller NanoString CodeSet Century [19], which consists of 89 OR genes and 11 reference genes, we developed ANOVA models that also dealt with assessing the DE genes relative to a FC threshold. The negative controls could not be included in these ANOVA models, as their residuals were making the general residual distribution heavily tailed and therefore non-Gaussian. Equality of variances was not met but within an acceptable borderline range on these genes. The results of these ANOVA models were not satisfactory, as the genes that were identified as DE genes (with FCs significantly higher than a certain predefined threshold) were sometimes very different from the TREAT, tTREAT and tTREAT2 results, and less biologically meaningful. ANOVA models including various genes in the same linear model can be applied on NanoString data and may be very useful for certain purposes. On our data, however, the gene-wise linear models and thus TREAT, tTREAT, or tTREAT2 were preferred over an approach that models all genes simultaneously.

## Conclusions

Our gene-wise statistical analysis of gene expression data with significance relative to a FC threshold gives reproducible and reliable results on NanoString data of odorant receptor genes, the largest gene family in the mouse genome. Because it is difficult to set a biologically meaningful FC threshold in advance, we have developed methods that provide guidance in determining a stable FC threshold (in order to minimize false discoveries and/or missed genes) or in avoiding this choice altogether. By applying a two-stage approach, a safety margin around the FC threshold can be set, which is beneficial in certain situations. Our running FC model identifies FC thresholds in an automated way, and is thus a more objective model leading to results that are more reproducible. This model is well suited for the problem of higher FC variances of lower expression values, thus avoiding a bias.

## Methods

### NanoString data

A NanoString experiment is performed using one or more NanoString cartridges, each containing 12 lanes. In each lane one RNA sample is assayed. The cartridge is scanned and imaged, resulting in digital readings (raw counts) for every barcode (gene) in every lane [18]. A macro can then be used to collect raw counts of cartridges of interest into an xls file. We imported these collected xls files into the R environment, version 2.14 [33]. Counts were processed to eliminate systematic experimental variability, differing amounts of input RNA, and variability in background, in three steps: first a normalization with respect to the geometric mean of the positive control spike counts, then a normalization with respect to the geometric mean of a group of five reference genes, and finally a background correction, which consists of subtracting the mean + 2*SD (standard deviation) of the negative control counts. Count values <1 were fixed to background level. We define a gene as informative if the median of the normalized NanoString counts in wild-type control mice is ≥100.

The FC for gene g can be estimated for any two samples as the ratio of the geometric mean of the normalized counts of first sample $\left({{\tilde{x}}_{g}}^{1}\right)$ over the geometric mean of the second sample’s counts $\;\left({{\tilde{x}}_{g}}^{2}\right):\mathrm{FC}=\frac{{{\tilde{x}}_{g}}^{2}}{{{\tilde{x}}_{g}}^{1}}$. As ratios are naturally heavily skewed,  *log*_2_(*FC*)  values, referred to as M values, are represented in most figures and analyses instead of the FCs. In MA plots, M values are plotted against the quantity A, $\left(\mathrm{lo}g_{2}\left(\sqrt[{2}]{{{\tilde{x}}_{g}}^{1}.{{\tilde{x}}_{g}}^{2}}\right)\right)$, which represents an average of the NanoString counts for that gene. In MC plots, M values are plotted according to relative gene order on the chromosomes.

### Usage of the limma package

A linear model is fitted to the NanoString expression data (digital counts) for each gene, which is key in the limma package [5,12]. We use the log_2_ of digital NanoString counts as expression data for every lane for the limma application. Thus for a set of l lanes (assumed independent), there is a log_2_ counts vector $y_{g}^{T}=\left(y_{g1},\cdots ,y_{\mathrm{gl}}\right)$ for every gene g. For the linear model on any gene g, we write: E[***y***_*g*_] = **X***ψ*_*g*_. The experimental design (i.e. which biological replicates belong to which RNA sample) is captured by the design matrix **X**, and  *ψ*_*g*_ represents the unknown vector of coefficients. What we are interested in, are certain contrasts, as given by the contrast’s matrix **C** (for example ***C***^*T*^ = (1, − 1) captures a two RNA group comparison): *β*_*g*_ = ***C***^***T***^*ψ*_*g*_. By fitting the linear model, the *ψ*_*g*_ will be estimated which then easily leads to the estimates of the  *β*_*g*_. Linear model theory also shows that $\mathrm{Cov}\left[{\hat{\psi }}_{g}\right]=\sigma^{2}V_{g}$, so the variance-covariance of the contrasts is deduced as follows: $\;\mathrm{Cov}\left[{\hat{\beta }}_{g}\right]=\sigma^{2}C^{T}V_{g}C$, for ease of notation let’s put ***C***^*T*^**V**_*g*_***C*** = **Z**_*g*_. Therefore, the student’s t-statistic for the contrasts in question is then typically written as follows: $t_{g}=\frac{{\hat{\beta }}_{g}}{s_{g}\sqrt{z_{g}}}$, where *s*_*g*_ represents the variance estimator of the unknown gene-wise variance.

### tTREAT

Let *τ* be the FC threshold as predefined and *M*_*g*_ the *log*_2_(*FC*) of a gene g. If we take the example of a two RNA group comparison, $M_{g}={\bar{y}}_{g}^{2}-{\bar{y}}_{g}^{1}$, i.e. the difference in arithmetic means of the log_2_ NanoString counts of the two groups. It can easily be seen how this comparison now comes down to a contrast when applying a linear model to gene g.

The idea of an analysis relative to a FC threshold is to test the following composite hypothesis: *H*_0_ : |*M*_*g*_| ≤ *log*_2_(*τ*) against *H*_1_ : |*M*_*g*_| > *log*_2_(*τ*). Thus, *H*_0_ is an interval of values for *M*_*g*_ rather than a single value, which is normally 0. McCarthy and Smyth [17] determined the exact (and conservative) p value for this *H*_0_ on t* (called TREAT). We reformulate their ideas for t instead of t* as follows: Let *t*_*obs*_ be the observed value of the t-statistic for a certain gene g, the p value now equals: *p* = *P*{|*T*| ≥ *t*_*obs*_ | *H*_0_}. As *H*_0_ is an interval, we may find an upper bound of this p value by choosing the element of *H*_0_ that is the most difficult to reject (hence conservative). So, let ${\hat{M}}_{\mathrm{obs}}$ be the observed value of ${\hat{M}}_{g}$ and choose $M_{0}=\min\left(\mathrm{lo}g_{2}\left(\tau \right),\left|{\hat{M}}_{\mathrm{obs}}\right|\right)$. Then the actual p value is bounded above by *p* ≤ *P*{|*T*| ≥ *t*_*obs*_ | *M*_*g*_ = *M*_0_}. Let *δ*$=\frac{M_{0}}{s_{g}\sqrt{z_{g}}}$ , then if *t*_*obs*_ ≥ 0, *p* ≤ 2 * [*P*{*T* > *t*_*obs*_ − *δ*| *M*_*g*_ = *M*_0_}] and if *t*_*obs*_ < 0, *p* ≤ 2 * [*P*{*T* < *t*_*obs*_ + *δ*| *M*_*g*_ = *M*_0_}]. In order to differentiate our test from t* and TREAT [17], we refer to it as tTREAT.

### tTREAT2

In this two-stage design, we reconsider the relative to a FC threshold idea at the point where the composite null hypothesis is formulated: *H*_0_ : |*M*_*g*_| ≤ *log*_2_(*τ*) against *H*_1_ : |*M*_*g*_| > *log*_2_(*τ*). The interval to which *H*_0_ applies, is thus *H*_0_ : − *log*_2_(*τ*) < *M*_*g*_ < *log*_2_(*τ*). A larger interval, called **I**, is now defined around *H*_0_, for example **I** : [−*log*_2_(*θ*), *log*_2_(*θ*)] with *θ* > *τ*. Then, in a first stage, called the stop or go stage, a 100% × (1 − *α*_*stage*1_)  confidence interval (CI) for *M*_*g*_ is defined as usual: Mg−tdf,1−αstage12.sgzg,Mg+tdf,1−αstage12.sgzg, where df stands for the residual degrees of freedom. If this 100% × (1 − *α*_*stage*1_)  CI for *M*_*g*_ falls strictly within **I**, it is decided to call the gene g as non-DE (stop), otherwise the gene g goes on to the second stage (go). For all the go genes, the second stage p value calculation is done exactly as for the tTREAT part above with regard to the interval defined by *H*_0_. This approach allows for more flexibility, particularly when there is concern that the chosen tTREAT FC threshold may be too high. In such a case, *θ* could be set to a high value, and a lower value, e.g. *θ* − 1 or *θ* − 0.1, could be chosen for *τ*. In a second stage, the type I errors of both stages, *α*_*stage*1_  (defining the CI) and *α*_*stage*2_ (significance of tTREAT analysis on go genes), can be chosen freely and may differ from each other. Here we have chosen *α*_*stage*1_ to equal 0.05. The value of *θ* (and thus the length of the interval **I**) and its difference with regard to *τ* allow for a safety margin around *τ*, thus helping to reduce false positives in noisy data while keeping the false negatives fixed or reducing them as well.

### Running FC model

By using a fixed FC threshold, genes with lower expression levels have a greater chance of being identified as DE because of their higher variance. In order to determine which FC threshold is appropriate for a certain expression level (or range of expression levels), we developed a running FC model. We used a model similar to [22] to find a potential FC cut-off function. Then we applied this function is to a number of ranges r of different expression levels, resulting in r different FC thresholds to use in any type of subsequently applied analysis. A step-by-step description of the running FC model is as follows.

1. Discrete relationship between FC and average gene expression levels:

Any two-by-two comparison of RNA groups is considered separately in the model. For simplicity, assume there are only two RNA groups as above. For the running FC model, the actual FC, $FC_{g}=\frac{{{\tilde{x}}_{g}}^{2}}{{{\tilde{x}}_{g}}^{1}}$ is calculated for every gene g. When FC_g_ is < 1, the reciprocal is taken. Then the *M*_*g*_ = *log*_2_(*FC*_*g*_)  values are plotted against the average expression levels (AR_g_) of the reference group (say group 1, in our case these are the control mice), $AR_{g}={{\tilde{x}}_{g}}^{1}$. Subsequently, the overall AR range is divided into bins of different widths but containing an equal number of genes. In each bin j (*j* = 1, ⋯ , *m*), the b%-percentile of the *FC*_*g*_ for all genes in that bin is determined and named *bp*_*J*_. All these *bp*_*J*_ can be visualized on the M-AR plot.

2. Fitting a continuous function:

As in [22], the equation *bp* = *a* + *b*/***AR*** (where **AR** stands for the median of the AR_g_ values in a certain bin j) seems to fit the data quite well. A least-squares fit of this equation is thus fitted to model the FC b-percentiles over various gene expression levels.

3. Defining the appropriate FC thresholds for various gene expression levels:

The model fit of step 2 is used to get actual FC threshold values for a number k of gene expression level ranges. In this case the bins of gene expression ranges are typically distributed more evenly over the full AR range, in contrast to the binning in step 1. For each of the i different ranges (*i* = 1, ⋯ , *k*) we apply the FC_i_, as defined by the model fit of step 2, to all genes in that specific range i of expression values as a FC threshold value for any kind of analysis (TREAT, tTREAT, or tTREAT2). The complete analysis is then referred to as the running FC model.

### Calculation of the Area Under the ROC curve (AUC)

The R open-source package pROC was used to calculate AUC values of statistical tests [34].

### Software for the tools

A zip file of a folder containing the functions in R code [33] to use the analytic tools developed here (tTREAT, tTREAT2, running FC model, and MA and MC plots) is available as Additional file 4. The folder also contains an R script as an example of how to apply these functions. The ReadMe file illustrates and explains how to use the tools.

### Availability of supporting data

The biological datasets (MK29, MK37 and MK38) supporting the results of this article are available in the NCBI Gene Expression Omnibus [35], and are accessible through GEO Series accession number GSE53876: http://www.ncbi.nlm.nih.gov/geo/query/acc.cgi?acc=GSE53876.

## Abbreviations

AUC: Area under the ROC curve; CI: Confidence interval; DE: Differentially expressed; FC: Fold change; FD: False discoveries; LFC: Limit fold change model; MG: Missed genes; qPCR: quantitative polymerase chain reaction; SAM: Significance analysis of microarray; TREAT: t*-tests relative to a threshold.

## Competing interests

The authors declare that they have no competing interests.

## Authors’ contributions

All authors participated in the design of the methods. EV performed all the statistical analysis and drafted the manuscript. MK collected the NanoString data. MK and PM revised the manuscript critically. All authors read and approved the final manuscript.

## Supplementary Material

Additional file 1 **False discoveries and missed genes for TREAT and tTREAT on simulated data, at p = 0.01.** (A) The positive y axis shows the average of the false discoveries, and the negative y axis shows the average of the missed genes for TREAT and tTREAT on 400 simulated datasets. The x axis shows 40 different percentages of DE genes (ranging from 1% to 40%) that is simulated in each case. The data are simulated with respect to a FC difference *ω* of 1.3 (up or down), and the FC threshold *τ* used for TREAT and tTREAT is also 1.3. The black percentages next to the gray and black bars of tTREAT represent the percentual decrease (prefixed with a minus sign) or increase (prefixed with a plus sign) in false discoveries or missed genes with repect to the reference, TREAT (depicted in cyan and blue). Significance is set at p = 0.01. (B) Similar to panel A but now the data are simulated with respect to a FC difference *ω* of 1.5, and the FC threshold *τ* used for TREAT and tTREAT is also 1.5. (C) Similar to panel A but now the data are simulated with respect to a FC difference *ω* of 2, and the FC threshold *τ* used for TREAT and tTREAT is also 2. Click here for file

Additional file 2 Mean false discoveries and missed genes, together with 95% prediction intervals on simulated data. Click here for file

Additional file 3 **Benefit of using the two-stage design in a stringent/non-stringent test situation.** (A) The positive y axis shows the average number of false discoveries, and the negative y axis shows the average number of missed genes, over 400 generated datasets for three tests relative to a FC threshold. The x axis shows 40 different percentages of DE genes (ranging from 1% to 40%) that is simulated in each case. Significance is set at p = 0.01. The DE genes are simulated with respect to a FC difference *ω* of 1.5. Here, the reference test is the original tTREAT with a FC threshold *τ* of 1.5, thus a test with *τ* = *ω* (in gray/black). The stringent test with a FC threshold *τ* of 2.5 is in cyan and blue. The tTREAT2 is in orange and red. (B) The positive y axis shows the average number of false discoveries, and the negative y axis shows the average number of missed genes, over 400 generated datasets for three tests relative to a FC threshold. The x axis shows 40 different percentages of DE genes (ranging from 1% to 40%) that is simulated in each case. Significance is set at p = 0.01. The DE genes are simulated with respect to a FC difference *ω* of 2.5. Here, the reference test is the original tTREAT with a FC threshold *τ* of 2.5, thus a test with *τ* = *ω* (in gray and black). The non-stringent test with a FC threshold *τ* of 1.5 is in cyan and blue. The tTREAT2 with FC thresholds 2.5/1.5 is in orange and red. Click here for file

Additional file 4 **Software.** A zip file of a folder containing the functions in R code to use the analytic tools developed here (tTREAT, tTREAT2, running FC model, and MA and MC plots). The folder also contains an R script as an example of how to apply these functions. The ReadMe file illustrates and explains how to use the tools. Click here for file
