# Supplementary material for: Statistical analysis of differential gene expression relative to a fold change threshold on NanoString data of mouse odorant receptor genes
Source: BMC Bioinformatics. 2014 Feb 4;15:39. doi: 10.1186/1471-2105-15-39 (PMC4016238; doi:10.1186/1471-2105-15-39)
Supplement: Additional file 1 — False discoveries and missed genes for TREAT and tTREAT on simulated data, at p = 0.01. (A) The positive y axis shows the average of the false discoveries, and the negative y axis shows the average of the missed genes for TREAT and tTREAT on 400 simulated datasets. The x axis shows 40 different percentages of DE genes (ranging from 1% to 40%) that is simulated in each case. The data are simulated with respect to a FC difference ω of 1.3 (up or down), and the FC threshold τ used for TREAT and tTREAT is also 1.3. The black percentages next to the gray and black bars of tTREAT represent the percentual decrease (prefixed with a minus sign) or increase (prefixed with a plus sign) in false discoveries or missed genes with repect to the reference, TREAT (depicted in cyan and blue). Significance is set at p = 0.01. (B) Similar to panel A but now the data are simulated with respect to a FC difference ω of 1.5, and the FC threshold τ used for TREAT and tTREAT is also 1.5. (C) Similar to panel A but now the data are simulated with respect to a FC difference ω of 2, and the FC threshold τ used for TREAT and tTREAT is also 2. [file 1471-2105-15-39-S1.pdf]

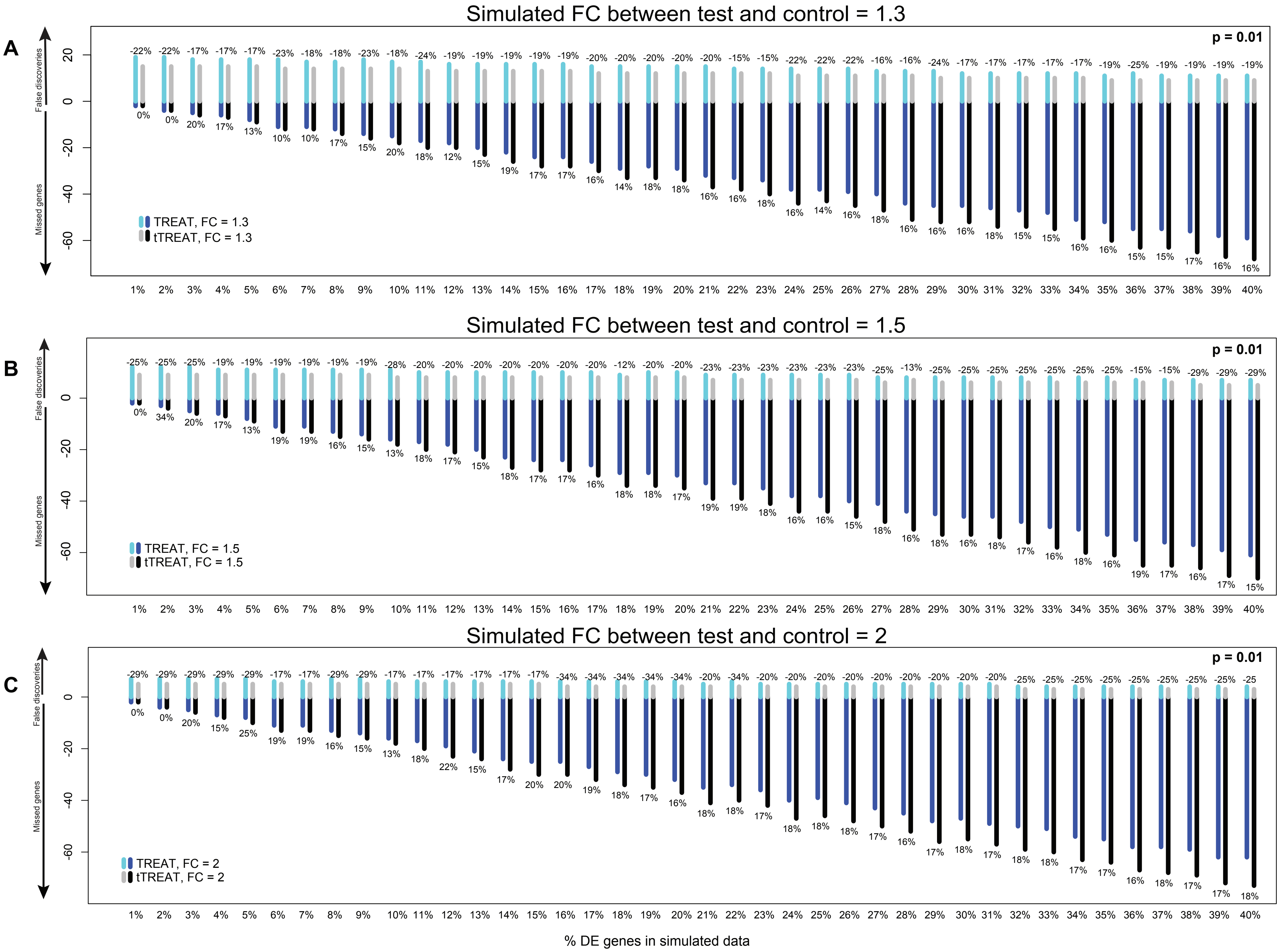
A
Simulated FC between test and control = 1.3
p = 0.01
False discoveries
Missed genes
20
0
-20
-40
-60
-22% -22% -17% -17% -17% -23% -18% -18% -23% -18% -24% -19% -19% -19% -19% -19% -20% -20% -20% -20% -20% -15% -15% -22% -22% -22% -16% -16% -24% -17% -17% -17% -17% -17% -19% -25% -19% -19% -19% -19%
0% 0% 20% 17% 13% 10% 10% 17% 15% 20% 18% 12% 15% 19% 17% 17% 16% 14% 18% 18% 16% 16% 18% 16% 14% 16% 18% 16% 16% 16% 18% 15% 15% 16% 16% 15% 15% 17% 16% 16%
TREAT, FC = 1.3
tTREAT, FC = 1.3
1% 2% 3% 4% 5% 6% 7% 8% 9% 10% 11% 12% 13% 14% 15% 16% 17% 18% 19% 20% 21% 22% 23% 24% 25% 26% 27% 28% 29% 30% 31% 32% 33% 34% 35% 36% 37% 38% 39% 40%
B
Simulated FC between test and control = 1.5
p = 0.01
False discoveries
Missed genes
0
-20
-40
-60
-25% -25% -25% -19% -19% -19% -19% -19% -19% -28% -20% -20% -20% -20% -20% -20% -20% -12% -20% -20% -23% -23% -23% -23% -23% -23% -25% -13% -25% -25% -25% -25% -25% -25% -25% -15% -15% -29% -29% -29%
0% 34% 20% 17% 13% 19% 19% 16% 15% 13% 18% 17% 15% 18% 17% 17% 16% 18% 18% 17% 19% 19% 18% 16% 16% 15% 18% 16% 18% 16% 18% 17% 16% 18% 16% 19% 17% 16% 17% 15%
TREAT, FC = 1.5
tTREAT, FC = 1.5
1% 2% 3% 4% 5% 6% 7% 8% 9% 10% 11% 12% 13% 14% 15% 16% 17% 18% 19% 20% 21% 22% 23% 24% 25% 26% 27% 28% 29% 30% 31% 32% 33% 34% 35% 36% 37% 38% 39% 40%
C
Simulated FC between test and control = 2
p = 0.01
False discoveries
Missed genes
0
-20
-40
-60
-29% -29% -29% -29% -29% -17% -17% -29% -29% -17% -17% -17% -17% -17% -17% -34% -34% -34% -34% -34% -20% -34% -20% -20% -20% -20% -20% -20% -20% -20% -20% -25% -25% -25% -25% -25% -25% -25% -25% -25
0% 0% 20% 15% 25% 19% 19% 16% 15% 13% 18% 22% 15% 17% 20% 20% 19% 18% 17% 16% 18% 18% 17% 18% 18% 18% 17% 16% 17% 18% 17% 18% 18% 17% 17% 16% 18% 17% 17% 18%
TREAT, FC = 2
tTREAT, FC = 2
1% 2% 3% 4% 5% 6% 7% 8% 9% 10% 11% 12% 13% 14% 15% 16% 17% 18% 19% 20% 21% 22% 23% 24% 25% 26% 27% 28% 29% 30% 31% 32% 33% 34% 35% 36% 37% 38% 39% 40%
% DE genes in simulated data
