# Supplementary material for: Statistical analysis of differential gene expression relative to a fold change threshold on NanoString data of mouse odorant receptor genes
Source: BMC Bioinformatics. 2014 Feb 4;15:39. doi: 10.1186/1471-2105-15-39 (PMC4016238; doi:10.1186/1471-2105-15-39)
Supplement: Additional file 2 — Mean false discoveries and missed genes, together with 95% prediction intervals on simulated data. [file 1471-2105-15-39-S2.pdf]

Additional File 2: mean false discoveries and missed genes, together with 95% prediction intervals on simulated data

FD* and MG$ results completing Figure 1

p = 0.05

Simulated FC between test and control = 1.3

		1% DE genes	10% DE genes	20% DE genes
TREAT, FC = 1.3	FD* [95%PI]	48 [34-63]	43 [30-57]	39 [27-52]
tTREAT, FC = 1.3	FD* [95%PI]	34 [23-46]	30 [20-41]	27 [17-38]
TREAT, FC = 1.3	MG$ [95%PI]	2 [0-5]	10 [2-19]	21 [9-34]
tTREAT, FC = 1.3	MG$ [95%PI]	2 [0-6]	12 [3-22]	25 [13-38]

Simulated FC between test and control = 1.5

TREAT, FC = 1.5	FD* [95%PI]	31 [21-42]	28 [17-39]	24 [14-35]
tTREAT, FC = 1.5	FD* [95%PI]	21 [12-30]	19 [11-28]	17 [9-26]
TREAT, FC = 1.5	MG$ [95%PI]	1 [0-4]	11 [3-20]	21 [10-33]
tTREAT, FC = 1.5	MG$ [95%PI]	1 [0-4]	13 [4-23]	26 [13-39]

1% DE genes 10% DE genes 20% DE genes

Simulated FC between test and control = 2

TREAT, FC = 2	FD* [95%PI]	18 [10-27]	16 [8-25]	14 [6-22]
tTREAT, FC = 2	FD* [95%PI]	12 [6-19]	10 [4-17]	9 [4-15]
TREAT, FC = 2	MG$ [95%PI]	2 [0-5]	11 [2-21]	23 [12-35]
tTREAT, FC = 2	MG$ [95%PI]	2 [0-5]	14 [4-25]	28 [15-42]

Simulated FC between test and control = 1.3

		1% DE genes	10% DE genes	20% DE genes
TREAT, FC = 1.3	FD* [95%PI]	19 [10-28]	17 [9-25]	15 [8-22]
tTREAT, FC = 1.3	FD* [95%PI]	15 [8-23]	14 [7-21]	12 [5-19]
TREAT, FC = 1.3	MG$ [95%PI]	2 [0-5]	15 [5-25]	30 [16-44]
tTREAT, FC = 1.3	MG$ [95%PI]	2 [0-5]	17 [6-28]	34 [19-49]

p = 0.01

TREAT, FC = 1.5	FD* [95%PI]	12 [5-19]	11 [5-17]	10 [4-16]
tTREAT, FC = 1.5	FD* [95%PI]	9 [3-15]	8 [3-13]	7 [2-13]
TREAT, FC = 1.5	MG$ [95%PI]	2 [0-5]	16 [6-26]	30 [15-45]
tTREAT, FC = 1.5	MG$ [95%PI]	2 [0-5]	18 [8-28]	35 [20-50]

1% DE genes 10% DE genes 20% DE genes

Simulated FC between test and control = 1.5

1% DE genes 10% DE genes 20% DE genes

Simulated FC between test and control = 2

1% DE genes 10% DE genes 20% DE genes

FD and MG results completing Figure 2

p = 0.01

TREAT, FC = 2	FD* [95%PI]	7 [2-12]	6 [1-11]	6 [2-11]
tTREAT, FC = 2	FD* [95%PI]	5 [1-10]	5 [1-9]	4 [0-8]
TREAT, FC = 2	MG[$] [95%PI]	2 [0-5]	16 [6-26]	32 [16-48]
tTREAT, FC = 2	MG[$] [95%PI]	2 [0-5]	19 [8-30]	37 [20-25]

Simulated FC between tets and control = 1.5 and Stringent test

		1% DE genes	10% DE genes	20% DE genes
tTREAT, FC = 1.5	FD* [95%PI]	10 [4-16]	8 [3-13]	8 [3-14]
tTREAT, FC = 2.5	FD* [95%PI]	1 [0-2]	1 [0-2]	1 [0-2]
tTREAT2, FC = 2.5/1.5	FD* [95%PI]	7 [2-12]	6 [1-11]	6 [1-11]
tTREAT, FC = 1.5	MG[$] [95%PI]	2 [0-6]	18 [7-29]	36 [21-51]
tTREAT, FC = 2.5	MG[$] [95%PI]	3 [0-7]	28 [15-41]	55 [36-74]
tTREAT2, FC = 2.5/1.5	MG[$] [95%PI]	2 [0-6]	19 [8-30]	36 [21-51]

FD and MG results completing Figure 3

p = 0.01

Simulated FC between test and control = 2.5 and non-stringent test

		1% DE genes	10% DE genes	20% DE genes
tTREAT, FC = 2.5	FD* [95%PI]	4 [0-8]	4 [0-8]	3 [0-6]
tTREAT, FC = 1.5	FD* [95%PI]	53 [35-71]	48[30-66]	42 [26-58]
TREAT2, FC = 2.5/1.5	FD* [95%PI]	48 [27-59]	43 [27-59]	37 [22-52]
tTREAT, FC = 2.5	MG[$] [95%PI]	2 [0-5]	20 [8-32]	38 [22-54]
tTREAT, FC = 1.5	MG[$] [95%PI]	1 [0-3]	10 [2-18]	20 [8-32]
tTREAT2, FC = 2.5/1.5	MG[$] [95%PI]	1 [0-3]	10 [2-18]	20 [8-32]

* FD = False Discoveries, the mean over 400 simulations

[$] MG = Missed Genes, the mean over 400 simulations
