# Supplementary figures and images for: Statistical analysis of differential gene expression relative to a fold change threshold on NanoString data of mouse odorant receptor genes
Source: BMC Bioinformatics. 2014 Feb 4;15:39. doi: 10.1186/1471-2105-15-39 (PMC4016238; doi:10.1186/1471-2105-15-39)

## Simulated FC between test and control = 1.5

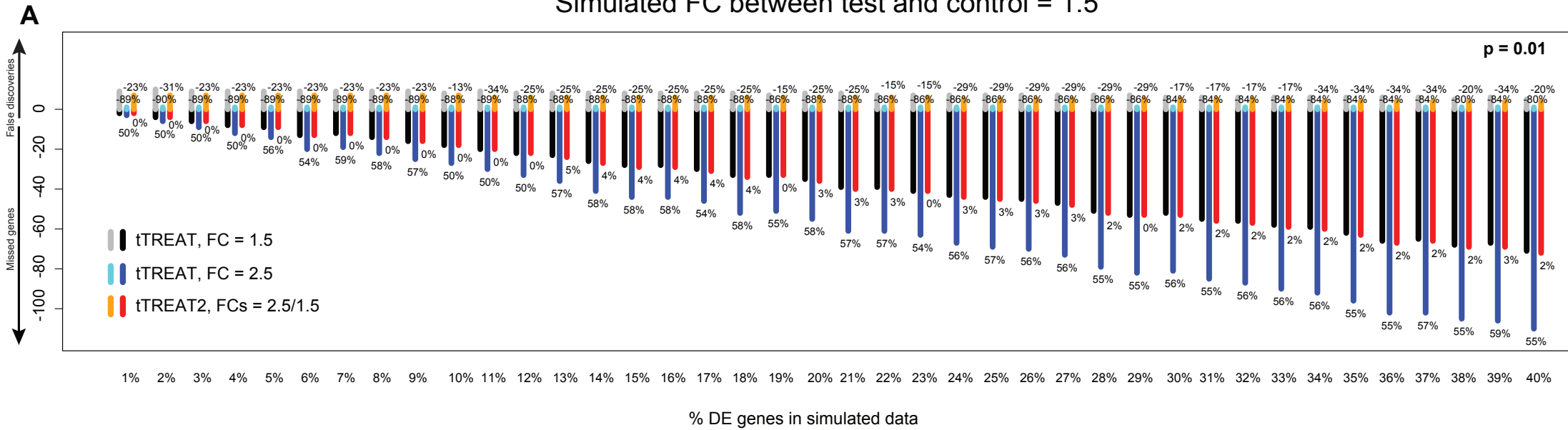

## Simulated FC between test and control = 2.5

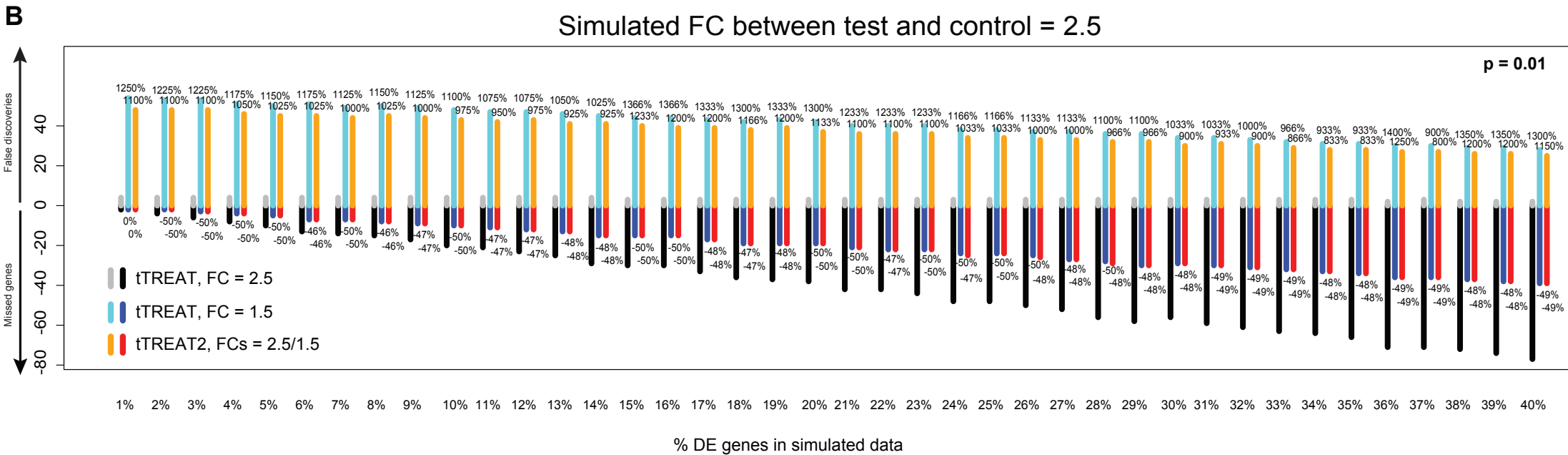

Supplement: Additional file 3 — Benefit of using the two-stage design in a stringent/non-stringent test situation. (A) The positive y axis shows the average number of false discoveries, and the negative y axis shows the average number of missed genes, over 400 generated datasets for three tests relative to a FC threshold. The x axis shows 40 different percentages of DE genes (ranging from 1% to 40%) that is simulated in each case. Significance is set at p = 0.01. The DE genes are simulated with respect to a FC difference ω of 1.5. Here, the reference test is the original tTREAT with a FC threshold τ of 1.5, thus a test with τ = ω (in gray/black). The stringent test with a FC threshold τ of 2.5 is in cyan and blue. The tTREAT2 is in orange and red. (B) The positive y axis shows the average number of false discoveries, and the negative y axis shows the average number of missed genes, over 400 generated datasets for three tests relative to a FC threshold. The x axis shows 40 different percentages of DE genes (ranging from 1% to 40%) that is simulated in each case. Significance is set at p = 0.01. The DE genes are simulated with respect to a FC difference ω of 2.5. Here, the reference test is the original tTREAT with a FC threshold τ of 2.5, thus a test with τ = ω (in gray and black). The non-stringent test with a FC threshold τ of 1.5 is in cyan and blue. The tTREAT2 with FC thresholds 2.5/1.5 is in orange and red. [file 1471-2105-15-39-S3.pdf]
