# Supplementary material for: Statistical analysis of differential gene expression relative to a fold change threshold on NanoString data of mouse odorant receptor genes
Source: BMC Bioinformatics. 2014 Feb 4;15:39. doi: 10.1186/1471-2105-15-39 (PMC4016238; doi:10.1186/1471-2105-15-39)
Supplement: Additional file 4 — Software. A zip file of a folder containing the functions in R code to use the analytic tools developed here (tTREAT, tTREAT2, running FC model, and MA and MC plots). The folder also contains an R script as an example of how to apply these functions. The ReadMe file illustrates and explains how to use the tools. [file 1471-2105-15-39-S4.zip › BMCBioInf.Code.OCT2013/ReadMe.pdf]

NSRFC = NanoString Relative to a Fold Change

Research Article BMC Bioinformatics: 'Statistical analysis of differential gene expression relative to a fold change threshold on NanoString data of mouse odorant receptor genes'

Information about the software

Evelien Vaes, Mona Khan and Peter Mombaerts
October 2013

What do you need before you can start?

1. Your R requires the following packages:

The correct functionality of our software depends on the following R packages (from bioconductor or cran):

Biobase
limma
multtest
SLqPCR

After you have downloaded the above-mentioned packages, the R script will load them into the R workspace.

2. The path of the directory where you have saved the unzipped folder:

This information is needed for instructing R where the functions of NSRFC are stored so that they can all be loaded into the workspace.

Example:
"/Users/evelien/Documents/TREATpackage/BMCBioInf.Code.OCT2013"

3. The path of the directory where your NanoString data are stored:

Using the nCounter RCC Collector Worksheet Excel macro provided by NanoString to import different RCC files, your data set will be assembled in one file as shown in Figure 1. Save this Excel file as a .csv file (comma separated values) without changing anything. Write down in which folder you have saved this .csv file, and its file name.

Note that you can define more than one data file (.csv) to be loaded, for instance if your experiment consists of more than one cartridge, or when you repeat the experiment later. The various data files will be joined into one R data matrix column-wise.

Example:
"/Users/evelien/Documents/TREATpackage/BMCBioInf.Code.OCT2013/Data" would be the directory and
"MKExample.Gorilla.24Sep13.csv" would be the name of the data file.

Now you can open the R script and set a few variables.

The R script: 'GeneralRScriptForNanoStringCartridge.Oct2013.R'

4. Open the R script

Open the R script entitled 'GeneralRScriptForNanoStringCartridge.Oct2013.R' and tell it where to find the functions and the NanoString data. For this, we have to name some of the R variables in the beginning of the script.

Note: All the R variables that the script defines and that you may want to change are followed by the # symbol.

As an example: We continue to use the directories and names given above. In the script put

```
# Pointing R toward the directory in which the NSRFC Functions are stored (where the folder that was unzipped is
# saved on your computer)

FunctionsDir <-
"/Users/evelien/Documents/TREATpackage/BMCBioInf.Code.OCT2013/Functions" #!!

# Pointing R toward the working directory in which the csv file or files that you want to load into R are stored:

DataFileDir <-
"/Users/evelien/Documents/TREATpackage/BMCBioInf.Code.OCT2013/Data"  #!!

# Now name here the csv file(s) you want to be loaded:

FilesToLoad <- c('MKExample.Gorilla.24Sep13.csv')  #!!
```

Figure 1

	A	B	C	D	E
1	nanoString TECHNOLOGIES		nCounter RCC Collector Worl		
2	Import RCC Files...		Delete Data		
3	**File Attributes**				
4	**File name**			20110303_MK	20110303_MK
5	**ID**			704	706
6	**Owner**			Mona	Mona
7	**Date**			3/3//11/11	3/3//11/11
8	**File Version**			1.6	1.6
9	**GeneRLF**			Gorilla_C742	Gorilla_C742
10	**Comments**				
11					
12	**Lane Attributes**				
13	**Lane ID**			1	2
14	**FOV Count**			1150	1150
15	**FOV Counted**			1131	1135
16	**Scanner ID**			DA45	DA45
17	**StagePosition**			1	1
18	**Binding Density**			0.69	0.97
19	**Messages**				
20					
21	**Reporter Counts**			129SvEv @ 21d	
22	*Code Class*	*Name*	*Accession*	704 +/+	706 +/+
23	Positive	POS_A(128)	ERCC_00117.1	32812	35070
24	Positive	POS_B(32)	ERCC_00112.1	8732	9661
25	Positive	POS_C(8)	ERCC_00002.1	2000	2265
26	Positive	POS_D(2)	ERCC_00092.1	632	700
27	Positive	POS_E(0.5)	ERCC_00035.1	109	138
28	Positive	POS_F(0.125)	ERCC_00034.1	72	84
29	Negative	NEG_A(0)	ERCC_00096.1	14	23
30	Negative	NEG_B(0)	ERCC_00041.1	18	19
31	Negative	NEG_C(0)	ERCC_00019.1	19	26
32	Negative	NEG_D(0)	ERCC_00076.1	18	25
33	Negative	NEG_E(0)	ERCC_00098.1	19	20
34	Negative	NEG_F(0)	ERCC_00126.1	13	4
35	Negative	NEG_G(0)	ERCC_00144.1	18	39
36	Negative	NEG_H(0)	ERCC_00154.1	33	47
37	Control	Omp	NM_011010.2	89450	105664
38	Control	Cnga2	NM_007724.2	93503	109030
39	Control	Adcy3	NM_001159537.	78943	89455
40	Control	Ano2	NM_153589.2	5112	5702
41	Control	Gnal	NM_177137.4	22367	25043
42	Control	Cnga4	NM_001033317.	36139	42969
43	Control	Gap43	NM_008083.2	35859	42943
44	Control	Gnas	NM_010309.3	58279	62449
45	Endogenous	Olfr1416	MOR103_3.1	333	383

5. Setting up the parameters of the analysis you wish to perform

At the beginning of the R script, you find some more parameters that can be changed, if you do not want to use the default values that we have given in the script.

DatStart = the integer number indicating at which row your actual data begins (so after the positive and negative controls and potential list of reference genes).

StrainFact = A factor indicating to which strain/cell type /group each column of your data matrix belongs. The control group should be alphabetically first. The order can be resolved easily by placing the letter ‘a’ in front of any name you want to give it.

HKgenes = Name the genes that are your reference genes. If you are not sure about a suitable list of reference genes and you want the geNorm (geNorm was developed in Vandesompele et al, ***Genome Biology***, 2002) algorithm to select them out for you, then define here the genes from which the geNorm algorithm should select.

geNorm01 = This takes only values 0 or 1. Put it to 1 if you would want the geNorm algorithm to pick the reference genes out of a list provided by you (in HKgenes), put it to 0 otherwise.

FCthres = The value of the Fold Change Threshold relative to which you would like to perform your TREAT or tTREAT analysis. If you want to perform tTREAT2, you can put this value to a vector of length 2, the first entry being the FC threshold value for the stop&go stage, the second entry being the FC threshold value for the last stage of the tTREAT2 analysis.

Pvalue = the p value significance you want to use in the TREAT/tTREAT/tTREAT2 analysis.

6. Running the first part of the script in the R console

Optionally, you can remove genes that have a median count on control mice < 100 from further analysis. If you wish to do so, include lines 114 to 129 into your code. If you do not wish to do so, comment these lines out and un-comment out line 131.

Then:
Everything up until the last section of the script (the following line):

```
# Actual tTREAT analysis and more interesting plots
#######################################################
```

can now be pasted in the R console and run.

Note: until here, you have been preparing R to know where the NSRFC functions are, to know where your data are, to load that data and to clean and normalize it.

Concerning the loading and normalizing of NanoString data: there is another alternative, the R cran package named NanoStringNorm (by Waggot et al, ***Bioinformatics***, 2012). This package can be used to load NanoString data, to perform the normalization and to do a lot of quality control on your NanoString experiment(s). When the normalized data object as created by NanoStringNorm is saved as a **matrix** with **row names set to the gene names**, the functions of the next section can directly be used on the data loaded and normalized by NanoStringNorm.

Statistical test relative to a FC threshold and some plots

7. The actual analysis relative to a FC threshold

There are 4 potential analyses (TREAT, tTREAT, tTREAT2 and running FC model) you can select to run:

Option 1: TREAT or tTREAT

The input parameter 'ModeratedStats' in the 'DEGenestTREAT' function allows you to select TREAT (put it to 'Y') or tTREAT (put it no 'N', this is the default)

Option 2: tTREAT2

Option 3: the running FC model

For this option, first a model is run that calculates the different FCs for different gene expression levels. This function, called LFC, has a lot of options (=input parameters) that are interesting to experiment with, such as 'PercFC', which indicates what percentile to use in each bin to model the relation between FC and expression level; or 'ModBins', which stands for the number of gene expression level bins in which a different FC threshold is to be estimated. More information about the LFC function and its input parameters can be found at the beginning of its R code file.

Copy the code of your selected option into the R workspace and run.
Note that you can run as many analyses as you want. The analysis that you selected can be repeated with different input parameters or you can compare the different tests relative to a threshold in case you don't know what your preferred one is.

8. Additional plots

For MC plots, you will need to have a data file that links the genes to the chromosome on which they reside. Perhaps you are interested in another ordering of the genes than the chromosomal, such as a numerical, alphabetical or gene family ordering. The MC plot does not necessarily expects a chromosomal ordering, but it can deal with any type of ordering as long as you provide a file that states the ordering variable (as a grouping variable) in the second column: It can be an external excel table or .csv file. We recommend that the data file has a row for each gene and two columns, the first column containing the gene names and the second column containing the chromosome numbers (or the letter 'X' for the X chromosome) or alternative ordering. We give an example in lines 200 and 202.

Depending on the analysis option you have chosen, you will have a results object entitled tTREAT.Res, tTREAT2.Res or tTREATRun.Res

Put the name of the results object you have generated in the following line (line 212) in the code:

```
RFCTest.Res <- tTREAT.Res  #!!
```

Finally copy the entire plot code into the R workspace and run. This will provide MA and MC plots for any comparison of n-1 groups against a control you are interested in.

In addition, the MA-plot function is capable of outputting a .csv file (in the data directory set at the beginning) with all the genes, their FC values and whether they are DE or not DE. Just check the MA-plot function code in the beginning where all input parameters are explained.

In case you have run a running FC model and you are interested in MA plots that also show the gene expression bins for which different FC thresholds have been applied, you can run an alternative MA plot as well. It's the commented-out line of code given by the following (line 218):

```
MAplot_RFC(x = ND.2g, ColTypes.factor = strains.2g,
RealDatStart = DatStart, DEList =
RFCTest.Res[[l]][which(RFCTest.Res[[l]][,'P.Val'] <
Pvalue),'GeneID'], rangePC = (1:6), rangeNC = (7:14),
OtherList = c(), addNames = 'Y', LFCList = Fconc.model)
```

9. Set the working directory back

In order to allow you to continue with a normal R functionality after working with this script, you may want to set the working directory back to what it was before working with this script. To do so, just copy that final line of the script and run.
